# Shared ancestry of algal symbiosis and chloroplast sequestration in foraminifera

**DOI:** 10.1126/sciadv.adi3401

**Published:** 2023-10-12

**Authors:** Doron Pinko, Sigal Abramovich, Eyal Rahav, Natalia Belkin, Maxim Rubin-Blum, Michal Kucera, Raphaël Morard, Maria Holzmann, Uri Abdu

**Affiliations:** ^1^ Department of Earth and Environmental Science, Ben-Gurion University of the Negev, Beer Sheva, Israel.; ^2^ National Institute of Oceanography, Israel Oceanographic and Limnological Research, Haifa, Israel.; ^3^ MARUM-Center for Marine Environmental Sciences, University of Bremen, Bremen, Germany.; ^4^ Department of Genetics and Evolution, University of Geneva, Quai Ernest Ansermet 30, Geneva 4 1211, Switzerland.; ^5^ Department of Life Science, Ben-Gurion University of the Negev, Beer Sheva, Israel.

## Abstract

Foraminifera are unicellular organisms that established the most diverse algal symbioses in the marine realm. Endosymbiosis repeatedly evolved in several lineages, while some engaged in the sequestration of chloroplasts, known as kleptoplasty. So far, kleptoplasty has been documented exclusively in the rotaliid clade. Here, we report the discovery of kleptoplasty in the species *Hauerina diversa* that belongs to the miliolid clade. The existence of kleptoplasty in the two main clades suggests that it is more widespread than previously documented. We observed chloroplasts in clustered structures within the foraminiferal cytoplasm and confirmed their functionality. Phylogenetic analysis of 18*S* ribosomal RNA gene sequences showed that *H. diversa* branches next to symbiont-bearing Alveolinidae. This finding represents evidence of of a relationship between kleptoplastic and symbiotic foraminifera.. Analysis of ribosomal genes and metagenomics revealed that alveolinid symbionts and kleptoplasts belong to the same clade, which suggests a common ancestry.

## Teaser

Phylogeny reveals a common origin for chloroplast sequestration and algal symbiosis in foraminifera.

## INTRODUCTION

A broad range of eukaryotic hosts acquired phototrophy by harboring algal endosymbionts or their chloroplasts (*1*). In foraminifera, a key phylum of protists, several lineages have independently acquired distinct symbiont lineages, including dinoflagellates, diatoms, cyanobacteria, unicellular Chlorophyta, and Rhodophyta (*2*, *3*). Kleptoplasty is another well-recognized mixotrophic strategy in foraminifera (*4*) that involves the sequestration of chloroplasts from algae while maintaining their functionality (*5*–*8*). This strategy evolved in diverse marine eukaryotes, including ciliates, dinoflagellates, foraminifera, saccoglossan gastropods, and flatworms (*1*, *2*, *9*, *10*). The prevalence of kleptoplasty in foraminifera from deep oceans to transitional water, from oxic to anoxic and from photic to aphotic habitats (*11*–*18*), points to a highly diverse functionality of the sequestered chloroplasts (kleptoplasts), which extend the host’s physiological flexibility (*4*, *19*–*22*). Thus, foraminifera present a highly accessible model to study the variable functions and forms of acquired phototrophy in single-cell marine eukaryotes.

Many documented cases of kleptoplasty in foraminifera are from species that inhabit aphotic environments. Several studies have shown that some species lack any detectable photosynthetic activity, thus indicating other functions of kleptoplasts in aphotic environments, such as sulfur and nitrogen uptake (*11*, *20*, *21*). The phototrophic activity of foraminiferal kleptoplasts was also documented by LeKieffre *et al.* (*23*), who used correlated TEM (transmission electron microscope) and nanoscale secondary ion mass spectrometry imaging to show carbon assimilation in foraminiferal kleptoplasts. Foraminifera also exhibit different retention times of kleptoplasts. Whereas some species retain them for only a few days, implying constant replenishment of their chloroplast reservoir (*24*), others can keep kleptoplasts functional for several months (*11*, *20*, *24*, *25*).

Kleptoplastic organisms obtain their chloroplasts from various algal lineages (*1*). In foraminifera, only diatom chloroplasts have been reported as kleptoplasts (*4*, *26*, *27*). Previous studies have shown that many kleptoplastic foraminifera exhibit morphological test adaptations such as protrusions/teeth, which are arranged around the aperture (*4*, *18*, *28*–*30*) and used for extracellular cracking of diatom’s frustules, and thus directly involved in chloroplast sequestration mechanisms (*4*, *6*, *18*, *28*, *29*). Specifically, electron microscopy studies have considerably advanced the knowledge of kleptoplasty in foraminifera by revealing their diatom source, the kleptoplast massive occurrences, and their life cycle within the host cell.

Until this date, foraminiferal kleptoplasty has been documented only in the order Rotallida occurring in five families (Elphidiidae, Haynesinidae, Nonionidae, Stainforthiidae, and Buliminidae) (*4*, *6*). Algal symbiosis is present in four major rotaliid families (Amphisteginidae, Asterigerinidae, Nummulitidae, and Calcarinidae) that are not phylogenetically related to kleptoplastic species. Algal symbiotic relationships have also been established in three miliolid families (Alveolinidae, Soritidae, and Peneroplidae) and in some planktonic foraminifera. A notable pattern is the high fidelity, in most cases, to a single clade of algal source (kleptoplasty) and partnership (photosymbiosis), which indicates a strong host specialization shaped by evolution, despite functional differences between these types of acquired autotrophy (*16*, *31*, *32*). In this study, we present the discovery of kleptoplasty in the benthic foraminifer *Hauerina diversa*, which belongs to the order Miliolida and thrives in coastal, oxygenated photic water in the eastern Mediterranean Sea.

## RESULTS AND DISCUSSION

### Miliolid foraminifera sequester chloroplasts

**Fig. 1.**
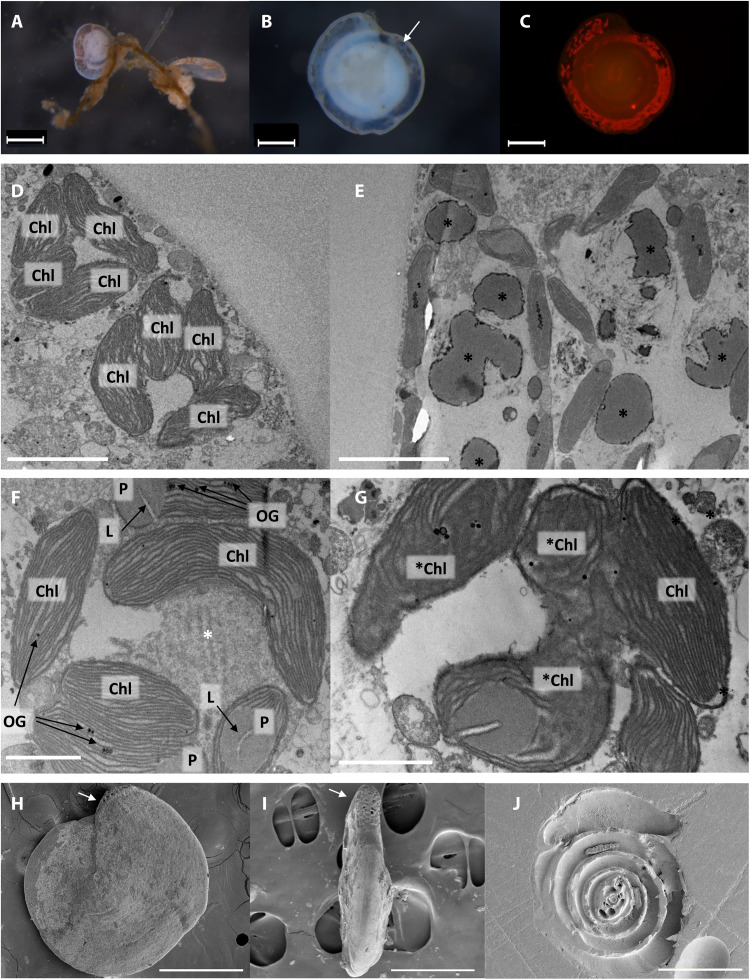
Visual appearance of the epiphytic benthic foraminifera *H. diversa* under stereoscope microscope, TEM, and SEM. (**A**) Specimen attached to macroalgae branch showing the epiphytic life mode (scale bar, 500 μm). (**B**) A living specimen under bright light showing abundant kleptoplast clusters that are visible in outer chambers. The white arrow points to one of the dark kleptoplast clusters. (**C**) Kleptoplast autofluorescence in the same specimen (red) following excitation at 480 nm. Scale bars, 200 μm (B and C). TEM micrographs (**D** to **G**) showing (D) chloroplast (Chl) in clusters arranged close to the cell surface; (E) chloroplast clusters that include dark vesicles (black *), possibly residues of the sequestrated diatom silicate envelope; (F) an evident diatom origin of the chloroplasts showing pyrenoid (P), lamella (L), and osmiophylic globules (OG) [note the typical appearance of chloroplast clusters enclosing an opaque body (white *)]; and (G) partially degraded chloroplasts (*Chl). Scale bars, 5 μm (D and E) and 2 μm (F and G). SEM micrographs showing *H. diversa* specimen, (**H**) *H. diversa* shell, and (**I**) edge view; white arrows point to *H. diversa* typical trematophore. *H. diversa* aperture is characterized by the trematophore, a sieve-like apertural structure. (**J**) Section of *H. diversa* shell. Scale bars, 500 μm (H) and 300 μm (I and J).

Our study reveals that kleptoplasty, the ability to sequester and maintain viable chloroplasts from an algal origin, has a broader phylogenetic range in foraminifera than previously documented. Kleptoplastic foraminifera are typically documented as infaunal (aphotic) species (*4*), with some reports of epiphytic species (*17*, *33*). Unlike many kleptoplastic foraminifera, *H. diversa* is epiphytic, living in a shallow photic environment attached to macroalgae (Fig. 1A).

Previous morphotaxonomic studies of *H. diversa* have not recognized its kleptoplastic nature, as they were mostly based on dead assemblages (*34*, *35*). In this study, live *H. diversa* specimens were retrieved from the Mediterranean coast of Israel. We observed dark granules in all *H. diversa* specimens with an autofluorescence signature, suggesting that chlorophyll-containing organelles consistently occur in *H. diversa* (Fig. 1, A to C). We further observed that the kleptoplasts move freely within the cell (Fig. 1C and movie S1) and that the host can control plastid movement, possibly in response to light, a fact that was also reported for other kleptoplastic species (*22*, *36*, *37*).

TEM micrographs revealed tight clusters of oval bodies containing dense, elongated thylakoid membranes (Fig. 1D). We compared these bodies to micrographs of other foraminiferal symbionts and noted that these clusters lack eukaryotic cell features such as a well-defined nucleus and external cell membrane containing the plastids (*26*). Thus, we conclude that these bodies are chloroplast clusters. Moreover, we examined the internal structures observed in these chloroplasts and noticed the typical architecture of diatom chloroplasts (Fig. 1F), such as the pyrenoid structure and the osmiophilic globules (*38*). These observations provided visual evidence for kleptoplasty in *H. diversa* and indicated their source to be from diatoms. The multiple chloroplast clusters observed in the micrographs revealed a tendency to be close to the cell surface (Fig. 1D).

**Fig. 2.**
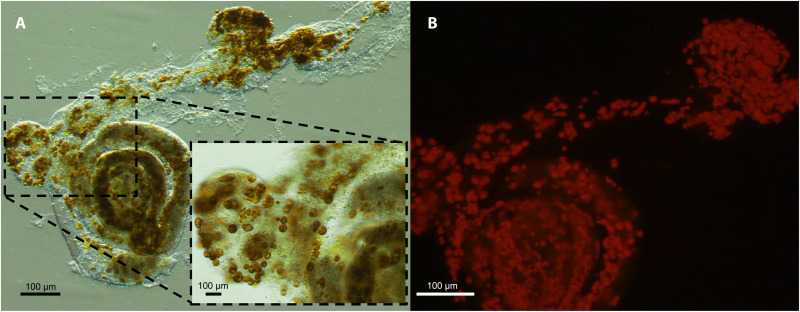
*H. diversa* kleptoplast clusters viewed in a decalcified specimen (removal of the calcite shell). (**A**) *H. diversa* dark kleptoplast clusters under bright field. (**B**) Kleptoplast cluster autofluorescence in the same specimen (red), following excitation at 480 nm.

The TEM micrographs showed that *H. diversa* kleptoplasts are grouped into clusters in various sizes containing several chloroplasts (Fig. 1, D to G). The chloroplast clusters can reach ~50 μm and are evenly distributed among the foraminiferal chambers (Fig. 2A). In addition, the kleptoplast clusters exhibit bright autofluorescence, indicating strong excitation of photosystem II, suggesting that these clusters contain viable organelles inside the cell of *H. diversa* (Fig. 2B).

The plastid “retention cycle” starts with the acquisition stage, marked by the appearance of dark bodies that seem to be a residue of diatom frustules (Fig. 1E), and ends with plastid degradation marked by disrupted thylakoids and pyrenoids (Fig. 1G). The dark bodies observed in the TEM micrographs (Fig. 1E) resemble the diatom frustules found in *Ammonia* sp. (*39*), indicating that the two species perform intracellular ingestion of the diatom silicate frustules. This strategy differs from the diatom sequestration process in the kleptoplastic foraminifers *Haynesina germanica* and *Elphidium williamsoni*, where the host collects its prey organelles through extracellular cracking of diatom frustules (*6*, *28*).

We assume that a sieve-like apertural structure known as the trematophore might play a role in chloroplast uptake by *H. diversa*, allowing it to crush the diatom frustules. This hypothesis is based on previous studies on kleptoplastic foraminifera, where “teeth”/tubercules structures were suggested to play a role in the diatom sequestration process (*4*, *6*). SEM (scanning electron microscope) micrographs showed the trematophore of *H. diversa* (Fig. 1, H and I) and the lack of internal functional structures, such as compartmentalization (Fig. 1J), that are typical for algal symbiont-bearing foraminiferal species (*26*, *40*). Examination of functional morphological features such as inner-shell architecture in symbiont-bearing foraminifera or tooth structures in kleptoplastic species is essential since it allows a better understanding of the foraminiferal adaptation to host diatoms or their plastids (*4*, *18*, *26*, *29*, *41*).

### Kleptoplasts contribute to *H. diversa* nutrition by photosynthesis

**Fig. 3.**
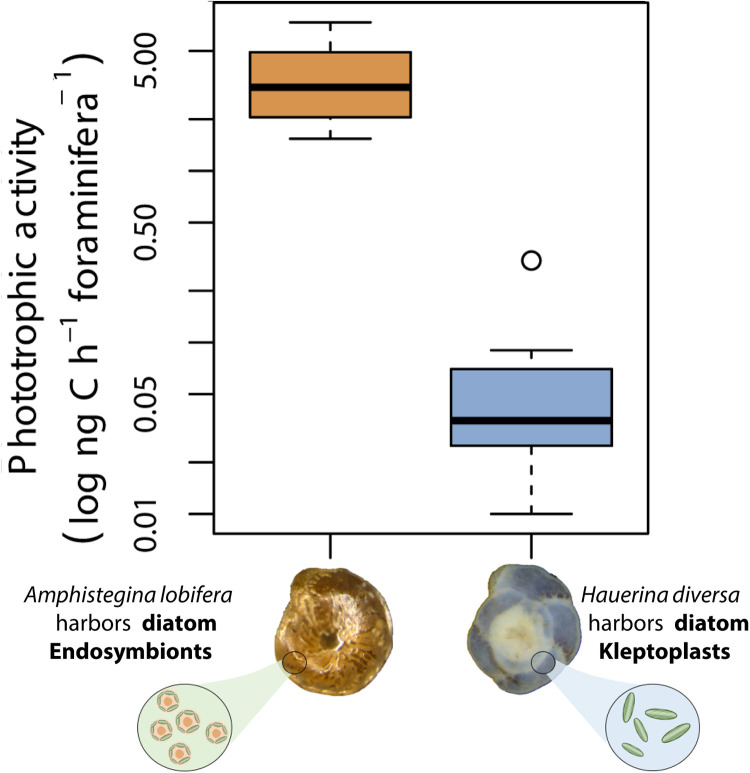
Carbon fixation rates (ng C hour^−1^ foraminiferal specimen^−1^) in *H. diversa* and *A. lobifera* calculated as the difference between specimens in light and dark incubation. The phototrophic activity shows substantial differences between the kleptoplasts of *H. diversa* and the diatom symbionts of *A. lobifera*.

*H. diversa* inhabits fully oxygenated and photic environments. Our carbon uptake experiment showed phototrophic activity of the kleptoplasts (ranged from 0.01 to 0.3 ng C hour^−1^ foraminifera^−1^; Fig. 3 and table S5) and thus proved their functionality. *H. diversa* photosynthetic activity per specimen was lower compared to the symbiont-bearing *Amphistegina lobifera*, which ranged from 1.54 to 7.34 ng C hour^−1^ foraminifera^−1^ (Fig. 3 and table S5). Similar differences in the photosynthetic activity were also observed between the kleptoplastic species *Elphidium crispum* and diatom symbiont–bearing *A. lobifera* and *Borelis schlumbergeri* (*15*). The absolute values of these experiments differ since our rates are expressed per foraminiferal specimen and not per foraminiferal biomass.

In the case of kleptoplasty, more of the photosynthetic products are available for the host than in algal symbiosis, where some of the products are retained by the algae. On the other hand, kleptoplasty requires regular replenishment of kleptoplasts, whose functionality might be suboptimal because the host might not be able to fully replace vital physiological molecules that are encoded in the algal nuclear genome.

### Kleptoplasty and diatom symbiosis evolved in sister foraminiferal clades

**Fig. 4.**
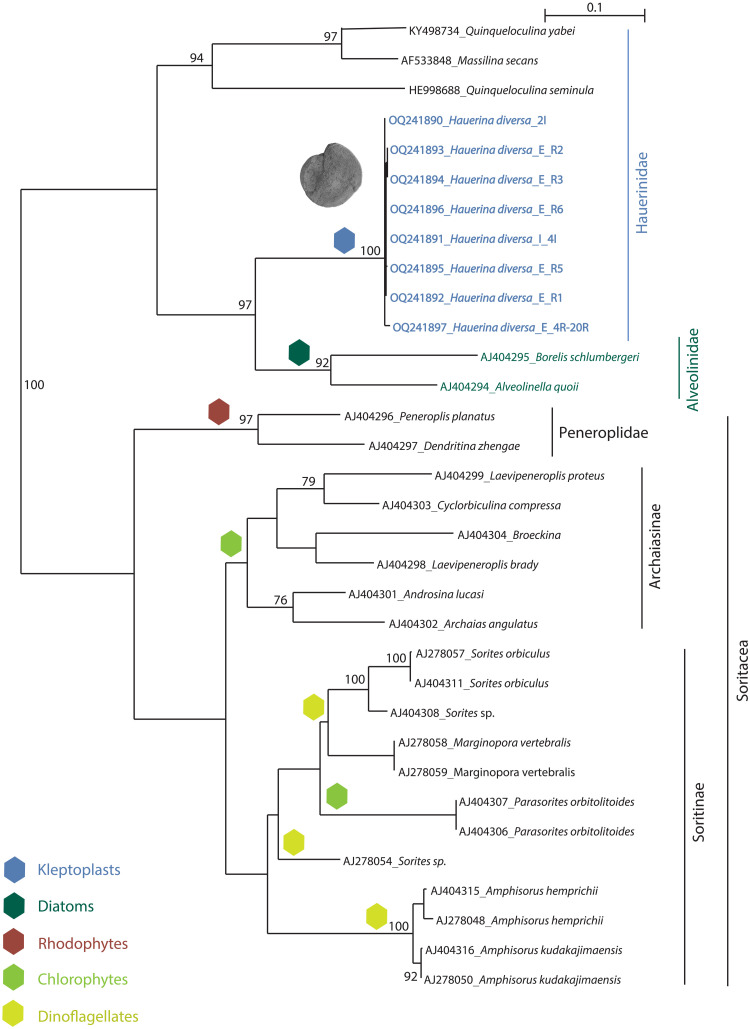
PhyML phylogenetic tree based on the 3′ end fragment of the SSU rRNA gene, showing evolutionary relationships of 33 miliolid foraminifera, representing all symbiont-bearing families. Symbiont type is indicated by color index. Taxa marked in bold are those for which sequences were acquired for the present study. The tree is unrooted. Specimens are identified by their accession numbers. Numbers at nodes indicate BVs. Only BV > 70% is shown; GTR substitution model with 1068 sites was used for analysis.

The foraminiferal sequences obtained for the present study cluster in two main clades (Fig. 4). One clade consists of kleptoplastic *H. diversa* [100% bootstrap value (BV)] branching as a sister to diatom-bearing Alveolinidae with strong support (97% BV). Three other species that are assembled in the family Hauerinidae (*Quinqueloculina yabei*, *Q. seminula*, and *Massilina secans*) form a monophyletic group (94% BV) that branches at the base of *H. diversa* and Alveolinidae. The second clade consists of three symbiont-bearing miliolid lineages. The Archaiasinae and Soritinae, which harbor chlorophytes and dinoflagellates, branch as sister groups with rhodophyte-bearing Peneroplidae at their base. The three groups are monophyletic, but only Peneroplidae is supported by high BV (97%). Molecular phylogenetic relationships of miliolid foraminifera were presented in a former paper (*42*), with Hauerinidae branching as a sister clade to Alveolinidae. Our obtained results showed that the type species of Hauerinidae, *H. diversa*, is branching as a sister to Alveolinidae, while the remaining Hauerinidae cluster at the base of this clade (Fig. 4), which results in a paraphyly of the latter family. The branching of *H. diversa* with the diatom-bearing Alveolinidae (Fig. 4) is, to the best of our knowledge, the first direct report of a close evolutionary relationship between a kleptoplastic and a symbiont-bearing lineage in foraminifera.

### Metagenomic data reveal abundant diatom plastids within *H. diversa*

**Fig. 5.**
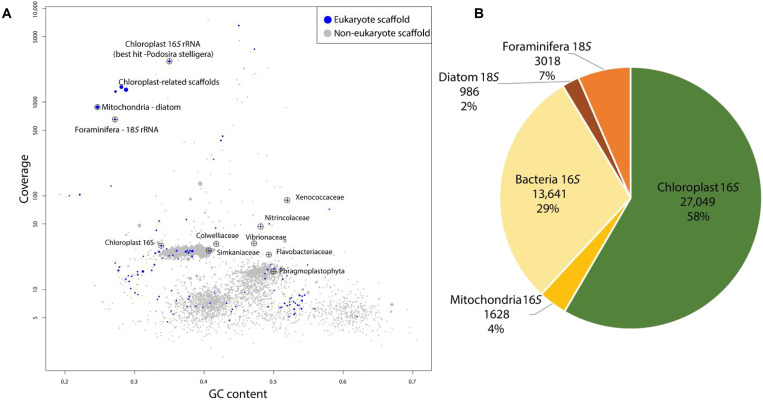
Metagenomic data reveal abundant diatom plastids. (**A**) Scaffolds of *H. diversa* metagenome grouped based on coverage and guanine-cytosine content. Each circle’s size corresponds to the length of the scaffold sequence. Crossed circles highlight the presence of SSU rRNA. Eukaryotic scaffolds are marked in blue. Note the highly abundant 12,706-bp scaffold containing the 16*S* rRNA gene of the chloroplast, with the best BLAST hit to the centric diatom *P. stelligera*. We could not assemble the host mitochondrion, yet its high-coverage fragments were found. (**B**) General taxonomic analyses of metagenomic reads of *H. diversa* that mapped to Silva138 database of 16*S* and 18*S* rRNA. The chloroplasts appear dominant in the dataset, accounting for almost 60% of the data.

Metagenomic data that were obtained from a pooled sample of 145 *H. diversa* specimens revealed that eukaryote organelles, in particular those of chloroplasts, were abundant in *H. diversa* (Fig. 5A). Chloroplasts were dominant, with circa 60% read abundance (Fig. 5B). They contained the 16*S* ribosomal RNA (rRNA) genes, with the best hit for melosirid *Podosira stelligera*, a centric benthic diatom that belongs to the subclass Coscinodiscophycidae (Fig. 5A). We were able to assemble a near-complete diatom mitochondrion, whose read abundance was lower than that of the chloroplast (Fig. 5). A less abundant nuclear diatom partial 18*S* rRNA gene sequence was also found. The mitochondrial and nuclear diatom DNA likely belongs to partially degraded diatoms. This is also associated with our TEM micrograph observation that indicates the presence of diatom chloroplasts without evidence of diatom cells containing distinct nuclei and mitochondria.

### A common origin of kleptoplasts and diatom symbionts points to a shared ancestry

Our results suggest a shared ancestry between kleptoplastic and diatom-bearing species in this lineage of foraminifera. Previous molecular studies revealed host-symbiont specificity for foraminifera with different kinds of symbionts living specifically in foraminifera (*43*–*45*). Although foraminifera harbor a large variety of photosymbionts, kleptoplasty has only been established with diatoms. The question remains open whether there is some advantage of incorporating chloroplasts from diatoms over other algal groups.

**Fig. 6.**
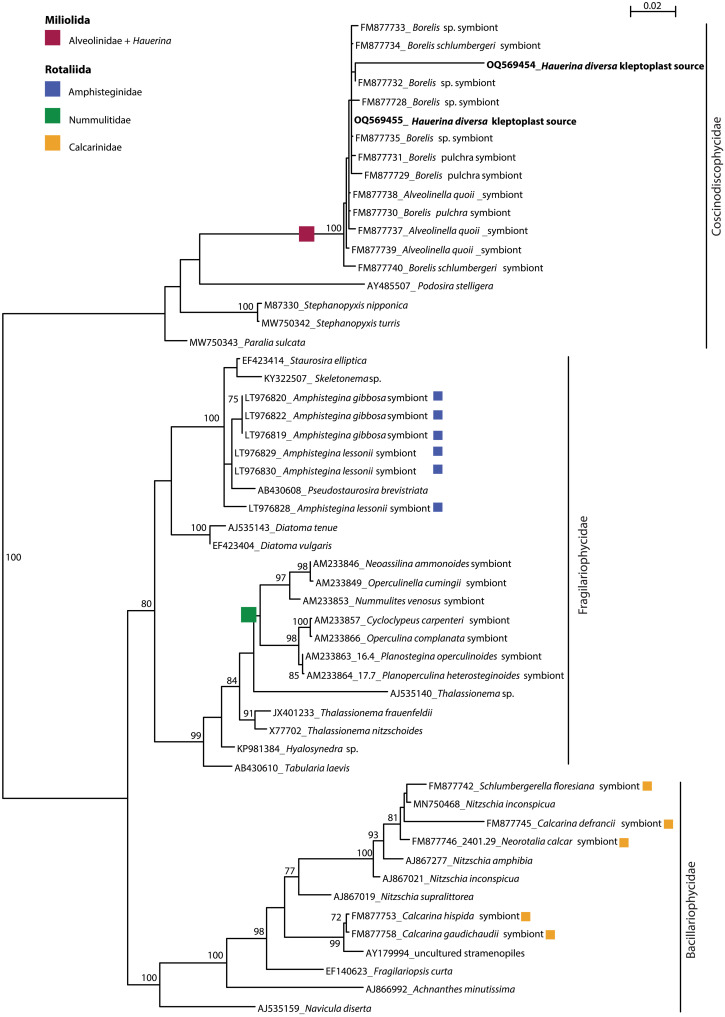
PhyML phylogenetic tree based on the SSU rRNA gene, showing evolutionary relationships of 54 diatom sequences belonging to three subclasses (Coscinodiscophycidae, Fragilariophycidae, and Bacillariophycidae). Diatom symbionts of foraminifera are indicated by their colors. The tree is unrooted. Specimens are identified by their accession numbers. Numbers at nodes indicate BV. Only BV > 70% is shown. TN93+R substitution model with 1874 sites was used for analysis.

The diatom 18*S* rRNA gene sequences cluster in three clades corresponding to the three subclasses Coscinodiscophycidae, Fragilariophycidae, and Bacillariophycidae (Fig. 6). Four rotaliid families of symbiont-bearing foraminifera—Amphisteginidae, Asterigerinidae, Nummulitidae, and Calcarinidae—established symbioses with distinct diatom clades (Fig. 6) (*45*–*47*). Amphisteginidae and Nummulitidae harbor Fragilariophycidae symbionts, whereas Calcarinidae harbor Bacillariophycidae, a sister clade to Fragilariophycidae. No sequence information about symbionts in Asterigerinidae is available. Miliolida host a diversity of symbiotic algae encompassing rhodophytes, chlorophytes, and dinoflagellates in Soritacea as well as diatoms in Alveolinidae (Fig. 4). Thus, Soritacea and Alveolinidae independently acquired algal symbionts along their evolution (*31*). Alveolinidae symbionts and *H. diversa* kleptoplast donors form a monophyletic group within Coscinodiscophycidae, with *Podosira*, *Stephanopyxis*, and *Paralia* branching at the base, indicating that the symbionts and the kleptoplasts have a common origin (100% BV; Fig. 6). This is the first documentation of phylogenetic proximity between kleptoplasts and endosymbionts in foraminifera and thus can indicate the existence of shared ancestry between them.

**Fig. 7.**
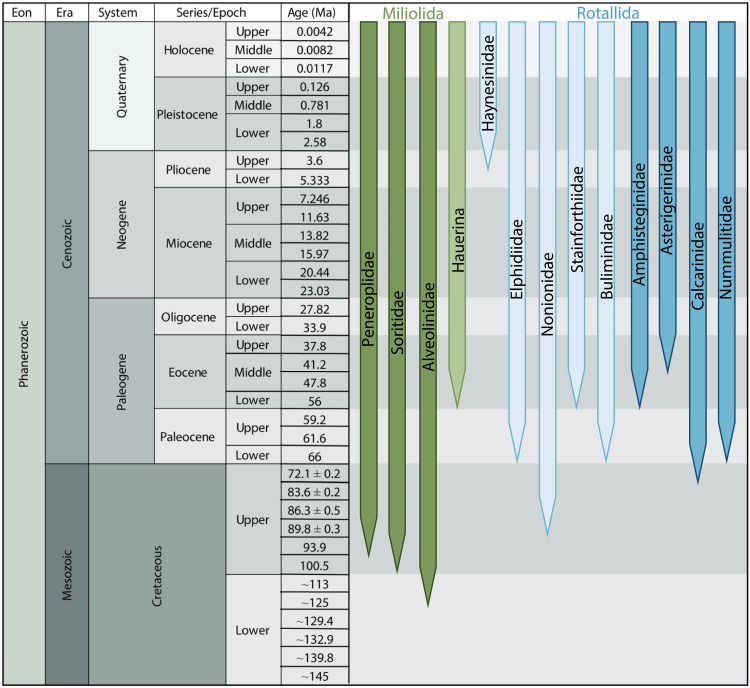
Modern foraminiferal mixotrophic lineages and their occurrence in the geological record, showing the independent evolution of both kleptoplasty (bright color) and symbiont-bearing (dark color) lineages in foraminifera.

Our study implies the existence of a shared ancestry for kleptoplasty and algal symbiosis in a lineage of miliolid foraminifera, revealed by the branching of *H. diversa* with the diatom-bearing Alveolinidae (Fig. 4) and the common origin of their algal source (Fig. 6). Our genetic results indicate that alveolinids and *H. diversa* share a common evolutionary ancestor (Fig. 4), which probably acquired synapomorphic traits for interacting with a specific group of centric diatoms. These traits might have later developed in one lineage (Alveolinidea) to algal symbiosis and in the other lineage (*H. diversa*) to a chloroplast sequestration strategy (Fig. 7). The link between kleptoplasty and algal symbiosis in foraminifera has a major outcome in our understanding of these strategies. Previously, kleptoplasty was described as an intermediate between algal symbiosis and emerging endosymbiotic events (*9*, *48*). Alternatively, this study suggests that kleptoplasty can represent a life strategy closer to algal symbiosis than previously understood.

## MATERIALS AND METHODS

### Collection and handling of *H. diversa* specimens

*H. diversa* is a Lessepsian immigrant that has been previously reported from the coastal Mediterranean Sea in macroalgal mats that cover the rocky environments (*34*). Macroalgae mats were scraped from the rocks at shallow depths (<0.5 m) and placed in sterile tubes/jars until they were brought to the laboratory (fig. S1). At the laboratory, the macroalgae were sieved through 250- to 2000-μm nets, and live specimens of *H. diversa* were handpicked under the stereoscope (125×) according to the distinct color of their kleptoplasts. The specimens were then placed in a petri dish with seawater to validate their vitality based on pseudopodial activity that was observed within a few hours. All specimens were imaged by a digital camera in a bright-field and fluorescent light for visual documentation of the kleptoplasts.

Live larger foraminifera used in the present study for phylogenetic analysis of symbionts were collected from the West Pacific (Japan and Indonesia), the Indian Ocean (Reunion and Maldives), the Red Sea (Israel), and the Atlantic (Bermuda and Florida Keys) (table S1). Sampling was carried out by dredging, scuba diving, or snorkeling. Living specimens were identified by brownish protoplasmic coloration due to endosymbiotic diatoms, isolated under a binocular, and cleaned with a brush before DNA extraction.

### DNA extraction, PCR amplification, cloning, and sequencing

Molecular phylogenetic analysis of *H. diversa* was done following Pawlowski and Holzmann (*49*) using the barcode region of the rDNA (ribosomal DNA) SSU (small subunit). Because of low DNA concentration, RNA was extracted from 3 to 10 specimens using the GENzol TriRNA Pure Kit. cDNA was produced using the Quantabio qScript cDNA Synthesis Kit. The cDNA was then amplified by seminested polymerase chain reaction (PCR) with primers 20R and 14F3 and reamplified with 20R and 14F1 (table S4). The thermal cycles for both PCR reactions were 3 min at 95°C, 20 s at 98°C, 15 s at 60°C, 30 s at 72°C, and 5 min at 72°C, repeating steps 2 to 4 35 times. The PCR product was run in 1.5% agarose gel, and only ~800–base pair (bp) bends were extracted from the gel and sequenced using Sanger. The obtained sequences were deposited in the National Center for Biotechnology Information (NCBI)/GenBank database.

For the present study, DNA was extracted from 21 large benthic Foraminifera belonging to Nummulitidae, Calcarinidae, and Alveolinidae (table S3 and Fig. 6) using the DNeasy Plant Mini Kit (Qiagen). Partial SSU rDNA of diatom symbionts was amplified following Holzmann *et al.* (*45*) with the primer pair diaf and diar (table S4). Thirty-five cycles were performed for the PCR, with an annealing temperature of 50°C. The amplified PCR products were purified using the High Pure PCR Cleanup Micro Kit (Roche Diagnostics) and cloned into competent *Escherichia coli* using the TOPO TA Cloning Kit (Invitrogen) following the manufacturer’s instructions. Sequencing reactions were performed using the BigDye Terminator v3.1 Cycle Sequencing Kit (Applied Biosystems) and analyzed on a 3130XL Genetic Analyzer (Applied Biosystems). The resulting sequences were deposited in the NCBI/GenBank database. Isolate and accession numbers are specified in table S1.

### Phylogenetic analysis

The eight obtained sequences of *H. diversa* were added to 25 miliolid sequences that are part of the publicly available 18*S* database of foraminifera (NCBI/Nucleotide; https://ncbi.nlm.nih.gov/nucleotide). All sequences were aligned using the default parameters of the Muscle automatic alignment option as implemented in SeaView version 4.3.3. (*50*). The alignment contains 33 sequences with 1068 sites used for analysis. The phylogenetic trees were constructed using maximum likelihood phylogeny (PhyML 3.0) as implemented in ATGC: PhyML (*51*). An automatic model selection by SMS (smart model selection) (*52*) based on AIC (Akaike information criterion) was used, resulting in a GTR substitution model being selected for the analysis. The initial tree is based on BioNJ. BVs are based on 100 replicates.

Symbiont sequences were added to 22 diatom sequences (table S2) that are part of the publicly available 18*S* database of Bacillariophyceae (NCBI/Nucleotide; www.ncbi.nlm.nih.gov/nucleotide/). All sequences were aligned using the default parameters of the Muscle automatic alignment option as implemented in SeaView version 4.3.3. (*50*). The alignment contains 54 sequences with 1874 sites used for analysis.

The phylogenetic tree was constructed using maximum likelihood phylogeny (PhyML 3.0) as implemented in ATGC: PhyML (*51*). An automatic model selection by SMS (*52*) based on AIC was used, resulting in a TN93+R substitution model being selected for the analysis. The initial tree is based on BioNJ. BVs are based on 100 replicates.

### Metagenomics analysis

DNA of *H. diversa* was extracted from 145 specimens using the DNeasy Plant Mini Kit (Qiagen). Metagenomics libraries were prepared at Syntezza (Jerusalem, Israel) and sequenced with 30 Gb on Illumina NovSeq at Novogene (Singapore). The assembly, annotation, and binning of the metagenome sequence data were done using the ATLAS pipeline (*53*). The raw sequences were assembled using SPAdes (*54*), using flags --meta -k 21,22,44,77,99,121, and mapped to the assembly using BBMap (B. Bushnell, sourceforge.net/projects/bbmap/) to produce guanine-cytosine content and coverage plots with gbtools (*55*). We used barrnap 0.9 (T. Seeman, https://github.com/tseemann/barrnap) to identify SSU 16*S* and 18*S* rRNA in metagenomic scaffolds. Eukaryote sequences were predicted using Whokaryote (*56*). The mitochondrial genome was annotated using the MITOS server (*57*), chloroplast open reading frames were identified using the NCBI ORFinder (https://ncbi.nlm.nih.gov/orffinder/), and NCBI BLAST was used to find the best hits to the sequences.

### Electron microscopy

SEM (HRSEM, Verios 460L) analyses were performed to examine *H. diversa* morphology and detect the presence of functional morphology structures. TEM analyses were done to characterize the morphological features of kleptoplasts and their positions within the cells of *H. diversa* and for preliminary identification of their algal source. The following procedure was done for the sample’s preparation: Fresh specimens of *H. diversa* were picked and fixed overnight with 2.5% glutaraldehyde (electron microscopy grade) in filtrated seawater buffer and then stored at 4°C. The test of each specimen was decalcified using 0.5 M EDTA (pH 8) for ~4 hours and washed with 0.5 ml of 0.1 M cacodylate buffer for 5 min three times. The cells were then postfixed with 2% osmium tetroxide (4% osmium tetroxide diluted in cacodylate buffer) for 1 hour and then washed with 0.5 ml of 0.1 M cacodylate buffer for 5 min twice. Next, the samples were incubated in 2% uranyl acetate for 1 hour in the dark and then washed with cacodylate buffer. Dehydration of the samples was done by rinsing in ethanol and acetone (table S2). After the dehydration process, the solution was slowly replaced with Epon (table S3). The samples were embedded in 100% Epon 812 inside flat molds and then polymerized in a 60°C oven for 48 hours. Ultrathin sections (70 to 90 nm) were cut using Microtome Leica UC7, placed on a grid, and stained with 2% uranyl acetate solution for 1 min. The micrographs were produced by TEM (JEOL JEM 2100F, Ilse Katz).

### Carbon uptake experiment

A labeled carbon (NaH^14^CO_3_) uptake was examined to evaluate the photosynthetic activity of the kleptoplasts in *H. diversa*. The carbon fixation was calculated as the delta between carbon uptake under light conditions and a dark blank. Diatom-bearing foraminifera (*A. lobifera*) were used as a “control” that enabled a comparison between diatom activity in full symbiosis (i.e., the whole diatom cell is endosymbiotically embedded within the host cell) to kleptoplasty by *H. diversa* (i.e., containing only chloroplasts). Three specimens from each species were incubated in Eppendorf tubes. Before the experiment, the specimens were cleaned by brushing in filtered seawater (0.22 μm) to remove epibiont algae and transferred to filtered seawater about 12 hours before the experiment began. The experiment started with a spike of 0.1 μCi NaH^14^CO_3_/ml [PerkinElmer, specific activity 56 mCi mmol^−1^, following Nielsen (*58*)] that was added to each Eppendorf containing 1 ml of 0.22-μm filtered seawater. The foraminiferal specimens were incubated in the laboratory under in situ light (15 to 23 μmol quanta m^−2^ s^−1^) at room temperature (~25°C) for 6 hours. Immediately after spiking, subsamples (50 μl) from randomly selected tubes were placed in scintillation vials added with ethanolamine to verify the levels of ^14^C that were effectively added (an “added activity” measure). After incubation, the seawater was gently removed. Fuming was done overnight in a sealed box containing an open tube of 32% HCl to remove excess inorganic carbon. Then, 1 ml of scintillation cocktail (Ultima-Gold) was added, and the radioactivity was measured using a TRI-CARB 4810 TR liquid scintillation counter. Blank samples were incubated in the same manner as the “light” samples only in the dark, and the reads by the scintillation counter were subtracted from the light specimen samples. Each obtained value was divided by three to normalize for a single foraminiferal specimen.

The autotrophic activity of foraminiferal kleptoplasts and endosymbionts was standardized per foraminiferal specimen as a conservative approach. We acknowledge that alternative normalization approaches, such as using the chlorophyll content or the size of the foraminifera specimen, could be used. However, chlorophyll content per cell may change based on physiological state rather than its biomass. Further, our use of radiolabeled bicarbonate for primary productivity measurements prevents us from using the same foraminiferal specimen to quantify chlorophyll content.
