# Supplementary material for: Shared ancestry of algal symbiosis and chloroplast sequestration in foraminifera
Source: Sci Adv. 2023 Oct 12;9(41):eadi3401. doi: 10.1126/sciadv.adi3401 (PMC10569721; doi:10.1126/sciadv.adi3401)
Supplement: Supplementary file 1 — Supplementary Text Fig. S1 Tables S1 to S5 Legend for Movie S1 [file sciadv.adi3401_sm.pdf]

Science Advances
AAAS

Supplementary Materials for

Shared ancestry of algal symbiosis and chloroplast sequestration in foraminifera

Doron Pinko *et al.*

Corresponding author: Sigal Abramovich, sigalabr@bgu.ac.il; Uri Abdu, abdu@bgu.ac.il

Sci. Adv. **9**, eadi3401 (2023)
DOI: 10.1126/sciadv.adi3401

The PDF file includes:

Supplementary Text
Fig. S1
Tables S1 to S5
Legend for Movie S1

Other Supplementary Material for this manuscript includes the following:

Movie S1

Supplementary Text

Transmission electron microscope (TEM) analyses were done to characterize the morphological features of kleptoplasts and their positions within the cells of *H. diversa* and for preliminary identification of their algal source. The following procedure was done for the sample's preparation: Fresh specimens of *H. diversa* were picked and fixed overnight with 2.5% GA/filtrated seawater buffer and then stored at 4°C until further process. The test of each specimen was decalcified using 0.5 mol/L EDTA pH 8 for ~4 hours until the cell was exposed. The EDTA was washed off with cacodylate buffer 0.1 M 0.5 ml for 5 min x3. The cells were then post-fixed with 2% osmium tetroxide (4% osmium tetroxide diluted in cacodylate buffer) for 1h and then washed with

cacodylate buffer 0.1 M 0.5 ml for 5 min x2. The samples were then incubated in 2% uranyl acetate for 1 hour in the dark and then washed with cacodylate buffer 0.1 M 0.5 ml for 5 min x2. Next, dehydration of the samples was done (Table S.1).

After the dehydration process, we slowly replaced acetone with Epon (Table S.2). The samples' tubes were placed in a tube rotator at a low speed during the Epon incubations.

The samples were embedded in 100% Epon 812 inside flat molds to assure proper orientation and then polymerized in a 60˚C oven for 48 hours. Ultra-thin sections (70-90 nm) were cut from the fixed and stained samples using a Microtome Leica UC7. The ultra-thin sections were placed on a grid and were stained with 2% uranyl acetate solution for 1 minute. The micrographs were produced by TEM- JEOL JEM 2100F Ilse-Katz.

Fig. S1.

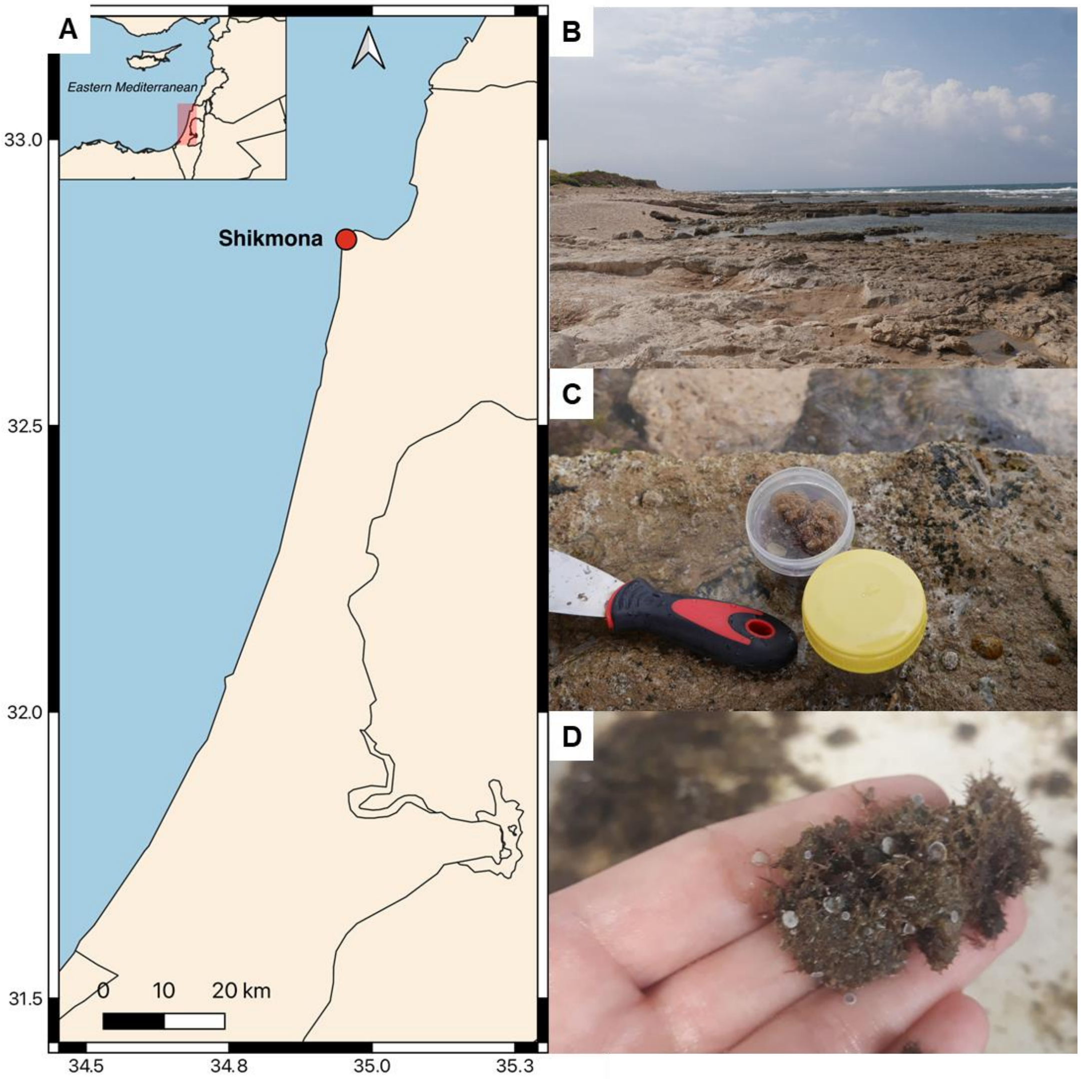

Figure S.1**: Sampling site location and sampling method.** (A) Illustration map showing the sampling site location (red dot), north of the Israeli coastline. (B) The rocky sampling site in Sikmona natural reserve, southern to the IOLR institute. (C) Macro algae were collected from beach rocks. (D) Foraminifera specimens on macroalgae, the most abundant large specimens are *Sorites orbiculus.*

Table S1.

Table S.1: Information on isolate, strain and accession numbers and sampling localities of foraminiferal host species and free-living diatoms. (n.a.: not applicable)

**Host species/Bacillariophyceae**	**Isolate/ Strain**	**Sampling locality**	**Accession number**
**Coscinodiscophycidae**			
Alveolinella quoii symbiont	298	Sesoko, Japan	FM877739
Alveolinella quoii symbiont	300	Sesoko, Japan	FM877737, FM877738
Borelis pulchra symbiont	747	Florida Keys, USA	FM877729, FM877730, FM877731
Borelis schlumbergeri symbiont	365	Elat, Israel	FM877734
Borelis schlumbergeri symbiont	629	Helengeli, Maldives	FM877740
Borelis sp. symbiont	191	Bermuda	FM877735
Borelis sp. symbiont	2405	St. Gilles, Reunion	FM877728
Borelis sp. symbiont	2406	St. Gilles, Reunion	FM877733
Borelis sp. symbiont	2407	St. Gilles, Reunion	FM877732
Hauerina diversa kleptoplast source	N12561	Israel, Mediterranean Sea	OQ569454
Hauerina diversa kleptoplast source	N3843	Israel, Mediterranean Sea	OQ569455
Paralia sulcata	CNS00428	Jiaozhou Bay, China	MW750343
Podosira stelligera	CCMP454	Wellington Harbor, New Zealand	AY485507
Stephanopyxis nipponica	CCMP817	Gulf of Maine, USA	M87330
Stephanopyxis turris	CNS00378	Jiaozhou Bay, China	MW750342
**Bacillariophycidae**			
Calcarina defrancii symbiont	866	Ishigaki, Japan	FM877704
Calcarina gaudichaudii symbiont	398	Sesoko, Japan	FM877758
Calcarina hispida symbiont	237	Sesoko, Japan	FM877753
Neorotalia calcar symbiont	2401	St. Leu, Reunion	FM877746
Schlumbergerella floresiana symbiont	2482	Bali, Indonesia	FM877703
Achnanthes minutissima	AMIN2	Luxembourg, Deifebaach river	AJ866992
Fragilariopsis curta	3	Antarctica, Mertz glacier region	EF140623
Navicula diserta	p750	Germany	AJ535159
Nitzschia amphibia	FDCC L602	Rush Lake, USA	AJ867277
Nitzschia inconspicua	NINC1	Ukraine	AJ867021
Nitzschia inconspicua	G5_2	Spain, Ebro Delata	MN750468
Nitzschia supralittorea	NZSU1	Luxembourg, Alzette river	AJ867019
uncultured stramenopiles	CCA42	Cape Cod, USA	AY179994
**Fragilariophycidae**			
Amphistegina gibbosa symbiont		USA, Florida, Tennessee Reef	LT976819
Amphistegina gibbosa symbiont		USA, Florida, Tennessee Reef	LT976820

Amphistegina gibbosa symbiont		USA, Florida, Tennessee Reef	LT976822
Amphistegina lessonii symbiont		Tansania, Zanzibar, Changuu Island	LT976828
Amphistegina lessonii symbiont		Tansania, Zanzibar, Changuu Island	LT976829
Amphistegina lessonii symbiont		Tansania, Zanzibar, Changuu Island	LT976830
Cycloclypeus carpenteri symbiont	659	Sesoko, Japan	AM233857
Neoassilina ammonoides symbiont	234	Sesoko, Japan	AM233846
Nummulites venosus symbiont	302	Sesoko, Japan	AM233853
Operculina complanata symbiont	20	Sesoko, Japan	AM233866
Operculinella cumingii symbiont	13	Sesoko, Japan	AM233849
Planoperculina heterosteginoides symbiont	17	Sesoko, Japan	AJ879135
Planostegina operculinoides symbiont	16	Sesoko, Japan	DQ440527
Thalassionema frauenfeldii	ECT3929ThalXL	USA, Gulf of Mexico	JX401233
Thalassionema nitzschoides	CCAP1084/1	Wales, UK	X77702
Thalassionema sp.	p474	Antarctica	AJ535140
Hyalosynedra sp.	AQ10	Taiwan, Kenting	KP981384
Tabularia laevis	s0021	n.a.	AB430610
Staurosira elliptica	L948	n.a.	EF423414
Skeletonema sp.	NMBguh008	n.a.	KY322507
Pseudostaurosira brevistriata	s0398	n.a.	AB430608
Diatoma tenue	L1251	n.a.	AJ535143
Diatoma vulgaris	L1535	n.a.	EF423404

Table S2.

Table S.2: Dehydration protocol of foraminiferal samples for electron microscopy. Single foraminifera specimens are placed in Eppendorf tubes and the solution is gradually replaced in each tube from cacodylate buffer to ethanol and then to acetone according to the stages described in the table.

Stage	Solution	No. of rinses	Duration
1	30% ethanol	2	10 min
2	50% ethanol	2	10 min
3	70% ethanol	2	10 min
4	95% ethanol	2	10 min
5	100% ethanol	2	20 min
6	100% acetone	3	5 min

Table S3.

Table S.3: Epon infiltration TEM protocol for foraminiferal samples. The sample solution is gradually replaced in each tube from Acetone to Epon according to the stages described in the table. (Note that in 100% Epon, the foraminifera cell is moved to a new tube using a pipette – do not touch the cell directly)

stage	Solution	No. of rinses	Duration
1	50% Epon812- 50% Acetone (0.5mL Epon812 & 0.5mL Acetone)	1	overnight
2	100% Epon812	2	2h
3	100% Epon812	1	overnight

Table S4.

Table S.4: Summary of primers specific for ribosomal barcode regions.

Objective	Gene targeted	Primer	Sequence	F	R	Reference
Phylogeny of *H. diversa*	Partial SSU 18S rDNA	14F3	ACGCAMGTGTGAAACTTG	X		Pawlowski et al. (58)
		14F1	AAGGGCACCACAAGAACGC	X		Pawlowski (59)
		20R	GACGGGCGGTGTGTACAA		X	
Diatom symbionts	Partial SSU 18S rDNA	diaf1	CGGAGAGGGAGCCTGAGA	X		Holzmann et al. (44)
		diar1	AGGGCATCACAGACCTGT		X	

Table S5.

Table S.5: Carbon uptake experimental results of both examined species: *Hauerina diversa* and *Amphistegina lobifera*, values are in ng C h^{-1} $foraminifera^{-1}$.

Sample number	*Hauerina diversa*	*Amphistegina lobifera*
1	0.01	1.54
2	0.02	1.64
3	0.02	1.70
4	0.03	2.41
5	0.03	2.68
6	0.03	2.73
7	0.04	3.41
8	0.05	3.84
9	0.06	4.46
10	0.08	5.37
11	0.09	7.27
12	0.30	7.34

Movie S1.

Short documentation of intracellular movement of the harvested chloroplasts cluster within *H. diversa*
